# Management Criteria of Grynfeltt's Lumbar Hernia: A Case Report and Review of Literature

**DOI:** 10.7759/cureus.3865

**Published:** 2019-01-10

**Authors:** Guglielmo Niccolò Piozzi, Riccardo Cirelli, Marco Enrico Mario Maino, Giovanni Lenna

**Affiliations:** 1 General Surgery, Università Degli Studi Di Milano, Milan, ITA; 2 General Surgery, Casa Di Cura Igea, Milan, ITA

**Keywords:** lumbar hernia, rare hernias, abdominal wall hernias, grynfeltt’s hernia, meshplasty, hernioplasty, case report, hernia repair

## Abstract

Grynfeltt's lumbar hernia is a rare abdominal wall pathology with around 300 cases described in the literature. Recently, a therapeutically aimed classification was proposed analysing the size, location, contents, muscular atrophy, origin, and existence of the previous recurrence. Surgical repair is the only definitive treatment option through either an open or laparoscopic approach. An 87-year-old female came to consult for swelling in the right lumbar area without traumatic history. A smooth, reducible, and tender mass of 4 x 3 cm was described. The suspicion of a Grynfeltt's hernia was confirmed by lumbar ultrasound with evidence of a 10 mm abdominal wall defect with the diameter increasing to 15 mm during a Valsalva maneuver. The patient had a primary type A lumbar hernia; therefore, open hernioplasty was performed. The patient was discharged from the hospital on the third postoperative day in optimal clinical condition. Her 12-month follow-up examination was uneventful.

A lumbar hernia diagnosis can be challenging. Preoperative imaging has an important role in assessing the size, location, and hernia contents. The use of a therapeutically aimed classification could be useful for optimal patient management and improvement of surgical outcomes.

## Introduction

The lumbar region is an anatomical area delimited superiorly by the 12th rib, inferiorly by the iliac crest, medially by the erector spinae muscles, and laterally by the external oblique muscle [1]. A lumbar hernia (LH) is defined as a protrusion of intraperitoneal or extraperitoneal contents through a defect in the posterolateral abdominal wall. Two weak triangular-shaped abdominal wall areas have been described in the lumbar region: the inferior and the superior lumbar triangle. The first was described by Petit in 1783 and is delimited by the iliac crest inferiorly, the latissimus dorsi muscle medially, and the external oblique muscle laterally [1]. The second was described by Lesshaft in 1870 and Grynfeltt in 1886 and is shaped like an inverted triangle (with the apex directed inferiorly) delimited medially by the erector spinae muscle, laterally by the internal oblique muscle, and superiorly by the 12th rib. The floor of Grynfeltt's hernia is constituted by the aponeurosis of the transversus abdominis and the roof by the latissimus dorsi muscle [1]. The superior LH can be considered a frank weak abdominal area because of no shielding action of the external oblique muscle on the transversalis fascia and because of the presence of the subcostal neurovascular bundle [2]. LHs are defined as diffuse in the case of considerable size that cannot be limited by the previously mentioned triangles. LH can be congenital (20%) or acquired (80%). The latter can be primary (75%) or secondary (25%) [2]. A primary LH can occur in the elderly, especially following severe weight loss [3]. Secondary LHs are associated with traumatic lesions or aortic, renal, or suprarenal surgery [4-6]. Congenital hernias arise from the inferior triangle, following erroneous embryological development, and are often associated with other anomalies as renal agenesis or a lumbocostovertebral syndrome [3, 7]. LH is most frequently unilateral, twice frequently on the left occurring between 5th and 7th decades with a M: F ratio of 2:1 [3, 8-10]. Bilateral LHs are rare and related to congenital defects [3]. The Grynfeltt triangle has been classified according to the variations in size and anatomy on a series of 50 adult human cadavers: type 0 (area: none; frequency 18%), type I (area: < 5 cm^2^; 50%), type II (area: 5 - 15 cm^2^; 22%), and type III (> 15 cm^2^; 10%) [1]. LH is frequently asymptomatic but may be associated with nausea, vomiting, renal deficiency, pain, and swelling in the lumbar region [11]. Diagnosis of an LH is basically clinical through palpation of the lumbar area with evidence of local swelling characterized by volume increase during a Valsalva maneuver [3, 8, 11]. However, imaging may be useful, especially in obese patients, for a precise anatomical evaluation of a hernia and an adequate differential diagnosis. Computerized tomography (CT) is considered to be the gold standard for the diagnosis and evaluation of the hernia content [12]. Thorek classified the LH according to the hernia contents into extraperitoneal (no peritoneal sac), paraperitoneal (peritoneum sliding and adhering to the viscera), and intraperitoneal (complete peritoneal sac around the visceral contents) [13]. In fact, the content of an LH may be retroperitoneal fat, kidney, colon, small bowel, omentum, ovary, spleen, or appendix [13]. Differential diagnosis must be made with lipomas, fibromas, hematomas, abscesses, kidney tumors, muscle hernia, panniculitis, and pannicular lumbosacroiliac hernia [14]. Surgical repair of an LH is the only definitive treatment option through an open or laparoscopic approach. Currently, synthetic meshplasty is the most popular among open repairs combined with muscle flaps depending on the nature of the defect [15]. We present a case of primary LH evidenced in our community institute.

## Case presentation

**Figure 1:**
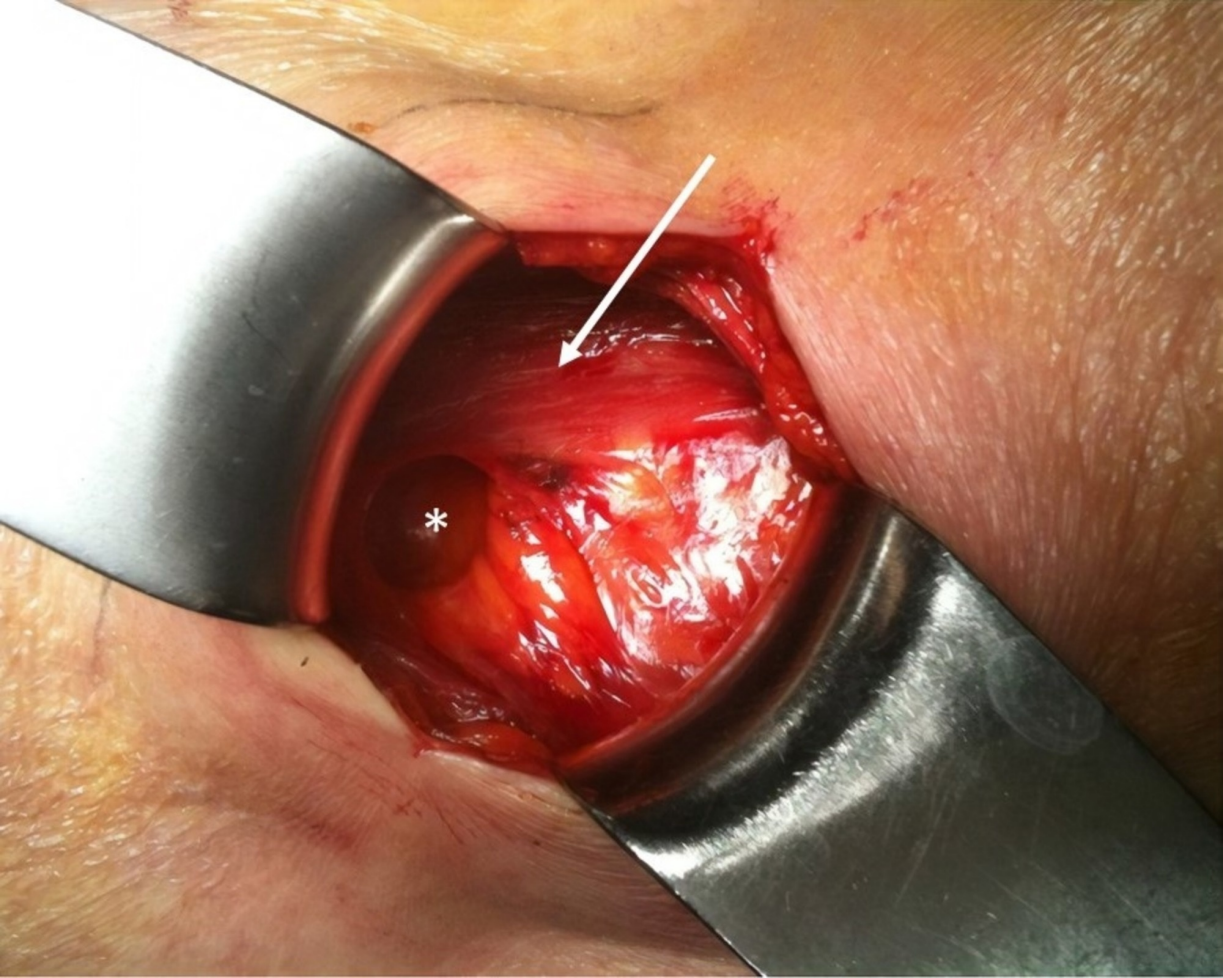
Intraoperative picture showing the defect after reduction of the hernia sac White star: hernia defect. White arrow: erector spinae muscle.

An 87-year-old female came to consult for swelling in the right lumbar area. The patient's past medical history was positive for a carotid aneurysm embolization, left hip prosthesis insertion, and multiple arthroses. A smooth, reducible, and slightly tender right lumbar mass approximately 4 x 3 cm was evident and a transmitted impulse could be felt during a cough. The lumbar swelling was reduced in the prone position. The suspicion for a Grynfeltt's hernia was confirmed by lumbar ultrasound (US) with evidence of herniation of a small intestinal loop through a 10 mm abdominal defect with a diameter increase to 15 mm during deep breathing. The patient was submitted to surgery under local anaesthesia. An open approach was performed. A lumbar transverse incision and a dissection of subcutaneous fat and the latissimus dorsi muscle fibres were performed in order to access the hernia sac (Figure 1). After reduction of the herniated mass, a direct suture was applied on the transversalis fascia. Reconstruction was performed with a polypropylene mesh with a circumferential overlapping of 3 cm in the extraperitoneal position. The mesh was fixed to the abdominal wall with interrupted non-absorbable monofilament sutures. The fibres of the latissimus dorsi muscle were approximated with loose absorbable sutures and the skin was closed with intradermal sutures. No drain was positioned. The postoperative course was regular. She was discharged on the third postoperative day in optimal clinical condition. Her 12-month follow-up examination was uneventful.

## Discussion

**Figure 2:**
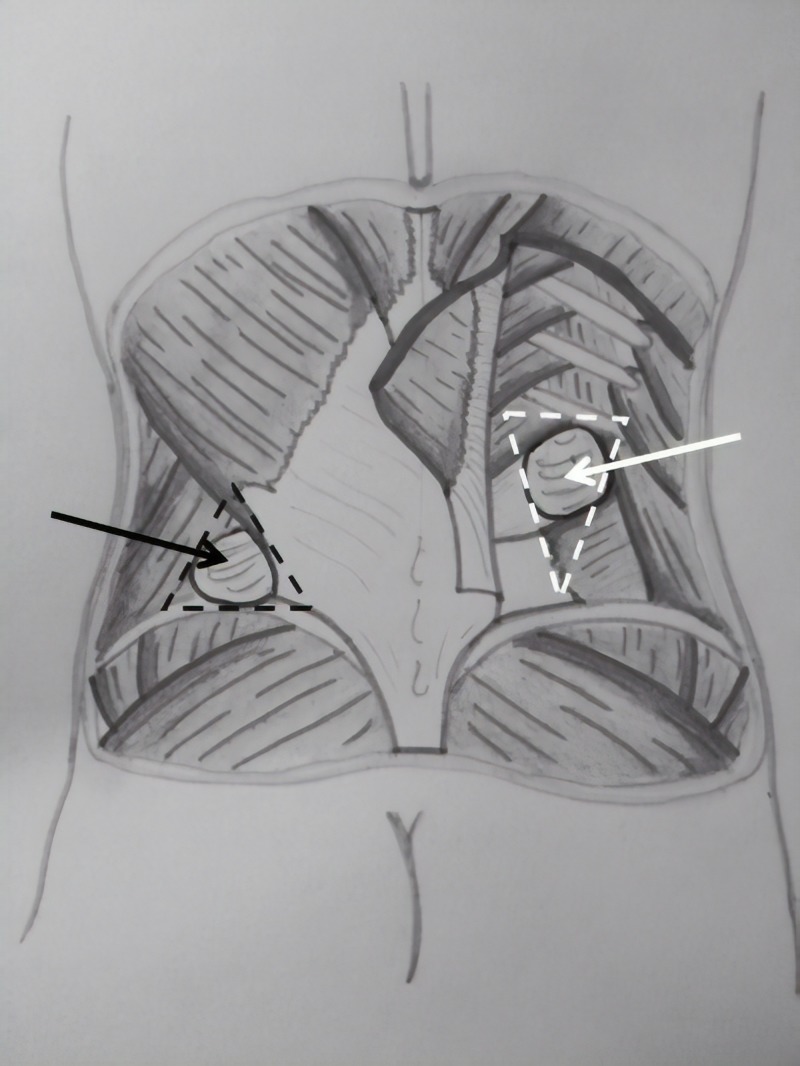
Grynfeltt's lumbar hernia and Petit's lumbar hernia Inferior (black dotted line) and superior lumbar triangle (white dotted line). Grynfeltt's lumbar hernia (white arrow) and Petit's lumbar hernia (black arrow).

LH is a relatively rare defect of the posterior abdominal wall with approximately 300 cases reported in the literature. LH can be divided into Grynfeltt's lumbar hernia and Petit's lumbar hernia (Figure 2). LH diagnosis and treatment can be challenging. Several unifactorial classifications have been proposed according to the location (superior, inferior, diffuse), the contents (extraperitoneal: with no peritoneal sac, paraperitoneal: peritoneum sliding and adhering to the viscera, or intraperitoneal: with a complete peritoneal sac around the visceral contents), and the aetiology (congenital or acquired) [11, 13]. Moreno-Egea et al. proposed the first therapeutically aimed classification system which identifies four types of LH based on six criteria: size, location, contents, muscular atrophy, origin, and the existence of previous recurrence [11]. The presence of at least two criteria is necessary for defining the LH type (Table 1) [11]. In the preoperative evaluation, we submitted the patient to lumbar US examination performed by a skilled radiologist who evidenced a herniation of a small intestine loop through a 10 mm abdominal defect. The increasing diameter (to 15 mm) during deep breathing, the quality of content, and the symptomatology were highly suspicious for a Grynfeltt's hernia; for these reasons and also because of the extreme age of the patient, we decided not to perform a preoperative CT scan. A skilled radiologist is of fundamental importance in order to exclude differential diagnosis and identify with higher specificity the characteristics and the contents of the LH. Several authors, such as Martin et al., underline the key role of preoperative routine CT scan for diagnosis and surgical planning [16]. Several techniques have been proposed in the literature; however, there are two that are the most frequently performed: an open approach with synthetic mesh placement and a laparoscopic approach. The open approach is the most common technique and is safe, effective, and not expensive. According to several authors [17-18], a laparoscopy may be considered as a safe and alternative method to an open approach. Moreover, laparoscopy improves the visualization of the abdominal wall defect and the relation with anatomical landmarks, avoids lumbar incision, allows the placement of a large mesh, and reduces postoperative pain and surgical site infection rates with faster recovery and better aesthetic results. In the present case, the patient had a primary type A LH with a dimension of 4 x 3 cm; therefore, we performed an open approach with a small lumbar incision. The postoperative course was normal, painless, and event-free. The patient was discharged on the third postoperative day in optimal conditions. At her 12-month follow-up, our patient denied any complications, such as pain, tenderness, numbness, or recurrence; therefore, the open approach can be feasible and safe in elderly patients. Our case report is fully compliant to the SCARE criteria (Surgical CAse REport Consensus Guidelines) [19].

**Table 1:** Classification of LH according to Moreno-Egea et al. [11] EP: extraperitoneal; IP: intraperitoneal; LH: lumbar hernia; LPS: laparoscopy; TEP: total extraperitoneal

Criteria	A	B	C	D (Pseudohernia)
Size (cm)	< 5	5 - 15	> 15	/
Location	Superior	Inferior	Diffuse	/
Contents	EP fat	Visceral	Visceral	/
Aetiology	Spontaneous	Incisional	Traumatic	/
Muscular Atrophy	No	Mild	Severe	Severe
Recurrence	No	Yes (open)	Yes (LPS)	/
Surgical Approach	Open (EP), TEP, LPS	IP LPS	Open	Open (double mesh)

## Conclusions

LH requires a careful evaluation for the correct operative management. Computerized tomography of the abdominal wall is considered the gold standard for the diagnosis and evaluation of the hernia content; however, in our opinion, a US examination performed by a skilled radiologist was more adequate in our present case. The use of a therapeutically aimed classification is crucial for a correct evaluation of the patient and the decision for the most suitable surgical approach.
